# The Power of Courts over Medical Witnesses

**Published:** 1860-10

**Authors:** 


*The Power of Courts Over Medical Witnesses,* has been recently determined by *Judge Marsh,* of Ohio, to the effect "that a medical witness could not be compelled to disclose facts confided to him in his professional capacity."

This is a very important step, if other and higher courts sustain the opinion. B.

